# A Brief Overview of Oxidative Stress in Adipose Tissue with a Therapeutic Approach to Taking Antioxidant Supplements

**DOI:** 10.3390/antiox10040594

**Published:** 2021-04-13

**Authors:** Shima Taherkhani, Katsuhiko Suzuki, Ruheea Taskin Ruhee

**Affiliations:** 1 Department of Exercise Physiology, Faculty of Sport Sciences, University of Guilan, Rasht 4199843653, Iran; 2 Faculty of Sport Sciences, Waseda University, 2-579-15 Mikajima, Tokorozawa 359-1192, Japan; 3 Gradute School of Sport Sciences, Waseda University, 2-579-15 Mikajima, Tokorozawa 359-1192, Japan

**Keywords:** oxidative stress, adipose tissue, obesity, antioxidant supplement

## Abstract

One of the leading causes of obesity associated with oxidative stress (OS) is excessive consumption of nutrients, especially fast-foods, and a sedentary lifestyle, characterized by the ample accumulation of lipid in adipose tissue (AT). When the body needs energy, the lipid is broken down into glycerol (G) and free fatty acids (FFA) during the lipolysis process and transferred to various tissues in the body. Materials secreted from AT, especially adipocytokines (interleukin (IL)-1β, IL-6, and tumor necrosis factor-α (TNF-α)) and reactive oxygen species (ROS), are impressive in causing inflammation and OS of AT. There are several ways to improve obesity, but researchers have highly regarded the use of antioxidant supplements due to their neutralizing properties in removing ROS. In this review, we have examined the AT response to OS to antioxidant supplements focusing on animal studies. The results are inconsistent due to differences in the study duration and diversity in animals (strain, age, and sex). Therefore, there is a need for different studies, especially in humans.

## 1. Introduction

Since 1998, the National Institutes of Health (NIH) has recognized obesity as a disease due to the impact of individuals’ health on society and the high economic and social costs incurred [1]. There is an adjacent link between obesity and metabolic disorders, including Alzheimer’s disease, respiratory problems, cardiovascular disease (CVD), type 2 diabetes (T2D), cancer, and non-alcoholic fatty liver disease (NAFLD) [2]. Body mass index (BMI) ≥ 30 has been accepted in many studies as one of the critical indicators of obesity. Still, this index is less valid than measuring the waist-to-hip ratio (WHR) due to the inability to count the lean body mass (LBM) [3].

Obesity is the result of overconsumption of nutrients and a sedentary lifestyle. As the consumption of nutrients increases, an imbalance is created between energy intake and expenditure, leading to fat accumulation in adipose tissue (AT) and obesity [4]. The World Health Organization (WHO) estimates the number of obese people globally at 650 million [2]. Several studies have shown that obesity depends on the regional distribution of excess body fat, not excess body weight. Thus, one of the most critical risk factors for obesity and related diseases is abdominal fat, which leads to the stimulation of pro-inflammatory and pro-oxidant states [5], the overproduction of free radicals, and pursuant oxidative stress (OS) in AT [6].

Scientists have made several efforts to control this disease. Various treatment methods, such as medication, surgery, exercise, and diet, have been considered in this regard. However, control of the disease is still far from expected. Increasing energy expenditure and subsequent weight loss is a smart way to control and prevent obesity [7]. In this regard, although authoritative articles have approved anti-obesity drugs, such as orlistat, and the use of weight-loss surgeries, the use of these methods is associated with many side effects [4]. Health researchers have identified diet, especially antioxidant supplements, as the most appropriate treatment for obesity [8]. Antioxidants affect the body’s endocrine and metabolic functions, leading to increased exothermic process and energy expenditure to reduce OS and body weight and improve obesity [9].

In this study, we have reviewed the effect of antioxidant supplements on AT changes under OS. The authors have allocated the content of this review article to the introduction, sources, and tools for measuring reactive oxygen species (ROS) in AT, a brief description of AT and related disorders, and finally, the relationship between antioxidant supplementation and obesity, respectively.

## 2. Overview of ROS

Living organisms need oxygen (O_2_) molecules to survive on earth. Therefore, these molecules’ presence is necessary to produce energy by the electron transfer chain (ETC) [10,11]. Under stressful conditions, O_2_ molecules in the body are converted into two separate atoms with unpaired electrons, named free radicals. These radicals are derived from O_2_ and are known as ROS [12]. ROS include superoxide anion (O_2_^•−^), hydrogen peroxide (H_2_O_2_), and hydroxyl radical (OH^.^), which play a vital role in causing pathophysiological damage, especially cellular damage to lipids, proteins, and deoxyribonucleic acid (DNA) [13,14,15]. When an unpaired electron is added to free radicals, O_2_^•−^ is formed. O_2_^•−^ has shown various behaviors in different environments. For example, in aqueous perimeters, this radical is reduced first to H_2_O_2_ by superoxide dismutase (SOD) and then converted to H_2_O and O_2_ by catalase (CAT). However, H_2_O_2_ may be converted to OH• in the presence of molecules containing ferrous iron (Fe^2+^) [16]. Various factors such as ROS concentration, time, and location of cells exposed to these species can determine the extent of these molecules’ damage. ROS is not harmful in low to moderate concentrations and has beneficial effects on cellular responses and signaling, gene expression, regulation of muscle power fluctuations, mitogenic responses, apoptosis, and protection against infections [11,12]. On the other hand, OH^.^ is highly reactive and harmful due to its very short half-life of only a few nanoseconds. Although H_2_O_2_ can be stable for a more extended period and does not damage cells, in higher concentrations, it has highly detrimental potency. H_2_O_2_ in aquatic environments, especially the human body, has a shorter half-life due to its neutralizing enzymes, which quickly cause irreparable damage to cells [17].

Since the discovery of ROS in 1970, most tissues in the body have been found to be affected by these reactive species, including cellular redox imbalance, OS, and cell dysfunction. ROS’s breakdown and production imbalance cause OS to alter cell function by damaging various molecules in the body [18,19]. In addition to ROS, other reactive species such as reactive nitrogen (RNS) and sulfur species (RSS) are also known as free radicals, although they are not derived from O_2_ [20]. When nitric oxide (NO) is added to O_2_^•−^, it creates a highly damaging radical named RNS that can cause the formation of peroxinitrate (ONOO^−^). This molecule causes nitrosative stress to various cells in the body [21]. When ROS is overproduced in the body, the antioxidant defense systems cannot eliminate or neutralize these species, and components such as proteins and lipids are damaged. Following this damage, pathological conditions such as vascular diseases (atherosclerosis, hypertension, and diabetes), respiratory disease, cell death, premature aging, neurological disorders, and degradation of skin enzymes (hyaluronidase and collagenase), platelet aggregation in vessels, and mutations and damage occur [22,23,24,25].

Many molecules play an essential role in maintaining the body’s homeostasis. However, one of the most important natural products of metabolism is ROS, which participate in numerous cellular signaling pathways in the body. Of course, these products have few effects on the cellular system, but their excessive production may have irreversible effects on the body’s various physiological systems [26]. The body uses endogenous antioxidant defenses against these molecules. However, in stressful situations, endogenous protection alone may not be enough to eliminate or neutralize ROS. In such cases, various exogenous factors such as diet, lifestyle, medication, and physical activity play an essential role in maintaining ROS balance [27]. One of the tissues that are severely affected by ROS imbalance is AT. Under similar conditions, adipokines secreted by AT such as leptin and adiponectin increase and decrease, respectively [16]. ROS production in AT occurs due to excessive consumption of nutrients [20,23,28]. It is noteworthy that the hormone adiponectin acts as an anti-inflammatory hormone in AT. Since obesity is an inflammatory disease, this hormone’s concentration in obesity decreases due to increased inflammatory cytokines. By reducing this hormone’s expression in obese people, its influential role in improving insulin sensitivity also diminishes. As a result, obese people face a complication named insulin resistance (IR), which predisposes them to T2D [29].

## 3. ROS Manufacturer Resources

**Figure 1:**
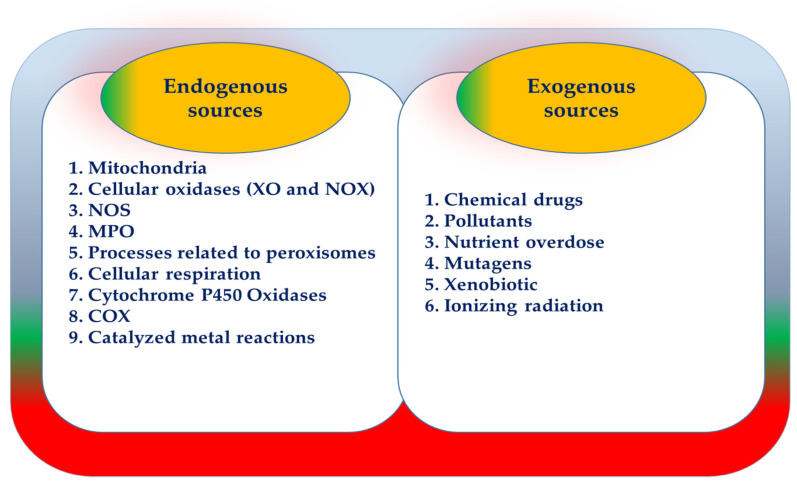
Reactive oxygen species (ROS) resources [24].

Multiple factors are responsible for the production of ROS, both endogenous and exogenous. Endogenous sources are: mitochondria, cellular oxidases (xanthine oxidase (XO), nicotinamide adenine dinucleotide phosphate (NADPH) oxidase (NOX)), nitric oxide synthase (NOS), myeloperoxidase (MPO), processes related to peroxisomes, cellular respiration, cytochrome P450 oxidases, microsomal cyclooxygenase (COX), and catalyzed metal reactions. ROS is also produced exogenously through sources such as chemical drugs, pollutants, nutrient overdose, mutagens, xenobiotics, and ionizing radiation (Figure 1) [24]. Several studies have shown that ROS-derived mitochondria and NOX are critical sources of ROS production in adipocytes [30].

### 3.1. ROS-Derived from Mitochondria

The main energy production source in the body is the mitochondria, which do this by oxidative phosphorylation. Interestingly, the O_2_^•−^ radical is mainly produced by oxidative phosphorylation. O_2_^•−^ is made in the mitochondrial ETC complex due to not being metabolized by about 0.15% and 2% of oxygen consumption in complexes I and III. Hence, mitochondria are one of the main sources of ROS and oxidative stress. After producing O_2_^•−^ mitochondrial manganese SOD (MnSOD) converts it to H_2_O_2_ [16,21]. On the other hand, ROS is mainly produced by the respiratory chain and during the formation of adenosine triphosphate (ATP). O_2_ is created by activating the oxygen molecule’s base state by transferring electrons or energy in the form of a single O_2_ [11].

### 3.2. NOX

Various cellular oxidases such as NOX and XO can produce ROS by reducing electrons from O_2_. Endothelial cells, chondrocytes, fibroblasts, myocytes, and phagocytes are the sites of NOX that produce ROS, particularly O_2_^•−^ and H_2_O_2_ to regulate cellular responses [31]. NOX initially produces O_2_^•−^, followed by produces H_2_O_2_ by the action of the antioxidant enzyme SOD. Scientists have confirmed that H_2_O_2_ at low concentrations can modulate the signaling pathway and metabolism and have a similar function to ATP and calcium (Ca^2+^). Because this radical crosses the cell membrane by aquaporins (AQPS) or proxy purines it can cause effects such as proliferation and recruitment of immune cells [32].

When germs attack these cells, NOX enzymes are activated during a respiratory burst. The enhanced products then absorb NADPH and O_2_. Thus, NADPH can act as an electron donor. This action starts the NOX enzyme complex in the plasma membrane by producing O_2_^•−^ from O_2_ molecules. In general, the production of O_2_^•−^ by NOX is related to the time when an electron is taken from NADPH in the cytoplasm and transferred to an O_2_ molecule [33].

NOX consists of a total of seven isoforms of catalytic subunits, including NOX 1-5 and dual oxidase 1 (Duox1) and dual oxidase 2 (Duox2). It should be noted that the main isoform of NOX in fat cells is NOX4. In response to the excessive consumption of glucose or palmitate, this isoform concentration in AT increases [21]. On the other hand, classical cytosolic subunits are not required for NOX4 activation, and only P22 ^phox^ is needed. Furthermore, the modulation of NOX4 activity is responsible for Polymerase delta-interacting protein 2 (Poldip2), which ultimately produces O_2_^•−^ and H_2_O_2_. NOX5 and Duoxs 1 and 2 do not require cytosolic subunits for activation. These three members of the NOX family must bind to intracellular N-terminal EF hand motifs via Ca^2+^ for activation. The EF hand has a helix-loop-helix structure, which is mainly found in calcium-bound proteins. This eventually leads to the production of O_2_^•−^ and H_2_O_2_, respectively [34,35,36,37]. In short, all NOX members except NOX5 need the P22 ^phox^ subunit to form. This subunit is usually regulated by the mineralocorticoid receptor (MR). It should be noted that all NOX components look at NADPH as an electron donor for the production of O_2_^•−^ and H_2_O_2_ [16]. NOX enzyme complexes play an important role in the production of O_2_^•−^ by transferring electrons from NADPH to O_2_. H_2_O_2_ is known as a highly absorbent radical in cell membranes. Finally, H_2_O_2_ is reduced to H_2_O and O_2_ by the enzyme CAT [34].

Mitochondria can produce ROS in both direct and indirect forms. Mitochondria can indirectly serve as a target for ROS production by the NOX enzyme complex, indicating a cross-link between NOX and mitochondria. In addition to acting as a potential source of ROS, mitochondria can also be responsible for NOX stimulation under certain conditions. This is especially important when ROS is neutralized by target mitochondrial antioxidant enzymes. By inhibiting ROS production, these enzymes can also partially alleviate NOX activity [33].

NADH and 1,5-dihydroflavin adenine dinucleotide (FADH2) are the products of glucose metabolism as electron donors in the tricarboxylic acid (TCA) cycle. This process eventually accelerates ROS production. On the other hand, the oxidation of free fatty acids (FFA) by mitochondria increases FFA intake. In this case, NADH and FADH2 are also produced by the oxidation of FFA-derived acetyl-CoA and the beta-oxidation of fatty acids (FAs) as electron donors. On the other hand, NOX is present on plasma membranes and can convert molecular O_2_ to O_2_^•−^. NOX may be closely related to ROS production associated with nutrient overdose [38]. Excessive FFA accumulation in adipocytes increases ROS production. On the other hand, ROS overproduction is reversed by NOX inhibitors such as diphenyleneiodonium or apocynin. This indicates NOX’s role in the production of ROS due to excessive consumption of fatty acids. Activation of NADPH oxidase by excessive consumption of fatty acids stimulates the synthesis of diacylglycerol and subsequent activation of protein kinase C (PKC) by FFA, especially palmitate [39]. FFA’s molecular mechanism that activates the NOX enzyme complex is closely related to the stimulation of diacylglycerol synthesis and subsequently activated PKC [21].

## 4. ROS Measuring Tools

ROS levels’ evaluation and measurement are important, practical steps to improve these reactive species’ effects. By measuring these species, a more accurate view of them can be achieved, and the appropriate treatment method can be used for each of them. Of course, direct measurement of ROS has its problems and difficulties [40]. This is important because some ROS, such as O_2_^•−^ and ^●^OH, while having very short half-lives of 5^−10^ and 9^−10^ s, respectively, also have very high reactivity. Over the years, countless indicators have been discovered to measure these species, but many of them did not provide consistent and reliable results and were easily discarded. However, in the following years, valid indicators were calculated to measure the oxidation of various tissues and cells of lipids, proteins, and DNA [41].

In general, the grouping of OS indicators is very important. In one group, the biochemical nature of molecules such as proteins, lipids, carbohydrates, and DNA is considered. While in the second group, products of oxidation of cellular compounds are formed to balance cellular mechanisms (oxidation-reduction). This group itself is divided into several subgroups [42]. The first group to free radicals leads to the change of various biomolecules such as malondialdehyde (MDA) from lipid oxidation, 4-hydroxy-2-nonenal (4-HNE), and reactive carbonyls from protein oxidation and 8-hydroxy-2’-deoxyguanosine (8-OHdG) of nucleic acid oxidation have been noted. In the second group, the relationship between free radical metabolism and physiological antioxidant defense molecules such as reduced glutathione (GSH) and CAT is considered. Furthermore, in the third group, modulation of free radicals with transcription factors such as c-Myc and Nuclear factor-κB (NF-κB) is important [16]. Here are the most common and commonly used indicators of OS:

### 4.1. MDA

When lipid molecules are exposed to OS, various products are produced in low-density lipoproteins (LDL) or cell membranes. One of its end products is MDA. The reactive substance, thiobarbituric acid reactive substances (TBARS), is responsible for measuring MDA levels [13].

### 4.2. 8-OHdG

When DNA is exposed to ROS, compounds such as 8-hydroxy guanine, 8-OHdG, and MDA-DNA are formed to break the DNA strands. 8-OHdG is composed of guanine oxidation, which plays an important role in mutagenic DNA damage and is used as a suitable indicator of oxidative damage to DNA [42].

### 4.3. 8-Nitroguanine (8-NO_2_-Gua)

In addition to producing 8-OHdG, guanine nitration also makes 8-NO_2_-Gua. These DNA metabolites are used to measure OS to DNA. In fact, in inflamed epithelial cells, 8-NO_2_-Gua levels increase. The amino acid polypeptides are separated by the reaction of amino acid side groups with ROS, and proteins are oxidized. With the oxidation of proteins, reactive carbonyl groups (aldehydes and ketones) are formed, and their tracing is known as an indicator of oxidative damage in protein molecules. It should also be noted that carbonyl groups are formed due to ROS reactions with proteins, carbohydrates, and lipids [43].

### 4.4. Oxidative Products of Sugars

Among the products produced by the oxidation of carbohydrates are advanced glycation end products (AGEs), which are formed due to non-enzymatic glycosylation of proteins. The highest presence of AGEs is in plasma and tissues, leading to diabetes, kidney failure, and aging [44].

### 4.5. Reduced Glutathione (GSH): Oxidized Glutathione (GSSG) Ratio

One of the most sensitive indicators of oxidative damage is the redox ratio GSH: GSSG. Influence on regulating gene expression, signaling conduction, NO metabolism, apoptosis, and impact on free radical scavenging are among the effective roles of GSH. On the other hand, the removal of ONOO^-^ is highly dependent on the formation of oxidized glutathione (GS-SG) by GSH, which is eventually converted to GSH through NADPH-dependent glutathione reductase [45]. Various signaling pathways such as C-Jun N-terminal kinase, protein kinase B, mitogen-activated protein kinase, apoptosis signal-regulated kinase 1, NF-κB, and protein phosphatase 1 and 2A are affected due to changes in the GSH/GSSG ratio [46].

## 5. A Brief Look at AT

The definition given to AT today is very different from what it used to be. It was previously thought that this tissue was just a tissue with the property of storing energy in the form of lipids. Today, a new perspective has emerged on it as an endocrine tissue [21,47,48,49]. Of course, the feature of this tissue’s storage source has helped many living things throughout history. When there is a lack or excessive consumption of nutrients, it has always been AT that has been able to help maintain the body’s energy homeostasis with hyperplasia or hypertrophy in different conditions [50,51,52,53].

On the other hand, the new look at this tissue owes much to discovering the hormone leptin in 1994 as a food controller. Even earlier, in 1987, it was found that sex steroids were metabolized in this tissue, followed by the production of adipsin [54,55]. Adipsin was one of the first adipokines to be identified in cultured adipocytes based on differentiation-dependent expression of its mRNA. Adipsin is an endocrine factor secreted by 3T3 fat cells [56,57,58]. Other important roles of adipose tissue in the body include effects on lipid and glucose metabolism, maintaining energy balance, appetite control, glucose homeostasis, insulin sensitivity, energy expenditure, inflammation, and repair of AT [59,60]. Various factors such as FFA supply, FFA esterification to triglycerides (TG), and TG degradation through the lipolysis process determine fat stores in AT. In general, the two enzymes of hormone-sensitive lipase (HSL) and adipose triglyceride lipase (ATGL) play an important role in the lipolysis process, which indicates the formation of FFA and glycerol as a result of the separation of the TG ester bond [61]. In general, AT contains high levels of stromal vascular cells, immunity, stem, endothelial, lymphocytes, adipocytes, preadipocytes, connective tissue matrix, and nerve tissue [55,62,63]. This tissue is also divided into brown adipose tissue (BAT) and white adipose tissue (WAT). There are apparent differences between BAT and WAT, morphologically. One of these differences is related to the size of the fat storage drops. White adipose cells are placed in a large fat drop (unilocular), and brown fat cells are placed in several small fat cytoplasm drops (multilocular) [64]. Another apparent difference between these two types of tissue is the number of mitochondria in them. The number of mitochondria in BAT is much higher than in WAT but the number of mitochondria in the WAT is limited. Because WAT plays an important role in lipid metabolism processes, including beta-oxidation and the TCA cycle, maturation, and differentiation of adipocytes, the importance of mitochondrial function is highlighted [16]. In this way, the BAT can maintain body temperature, especially when it is cold. BAT owes this feature to the high number of mitochondria within it. Of course, this tissue also plays a very important role in lipid oxidation [65].

The mitochondria’s inner membrane hosts a protein called uncoupling protein 1 (UCP1) that can generate heat by transferring protons to the mitochondrial matrix and separating oxidative phosphorylation and the electron transfer chain from ATP synthesis [60,66,67]. In fact, by consuming too many nutrients and being exposed to cold temperatures, UCP1 is expressed to protect the body’s organisms against obesity and the cold. Sympathetic neurons stimulate UCP1 inside the BAT to lead to exotherm and energy loss. Thus, in both humans and rodents, the association between obesity and UCP1 expression is inverse [68]. Sesterins are among the vital proteins associated with obesity due to oxidative stress, which play an important role in regulating metabolic homeostasis, suppressing ROS accumulation, and regulating the AMP-activated protein kinase (AMPK)-mammalian target of rapamycin complex 1 (mTORC1) signaling pathway. However, reducing these proteins in the body is associated with obesity and other metabolic disorders [69]. In general, three different isoforms have been discovered for sestrin (sestrin 1–3), of which sestrin 2 is more expressed in liver and adipose tissue. It has been estimated that sestrin 2 can reduce fat accumulation in AT and improve metabolic homeostasis by suppressing ROS and mTORC1 [70].

The most important site for cold-induced exotherm in rodents is BAT. This tissue is also involved in the exotherm of fat tissue. The distribution of BAT in humans and rodents varies according to their age. In humans, there is a large amount of BAT in the body only in early infancy, and with age, the distribution of BAT in the body decreases. However, in the case of rodents, the opposite is true. Because BAT expands as rodents live longer, on the other hand, different types of BAT have been deposited in the body in a scattered manner; interscapular BAT (IBAT) is the most important and vital type [71]. The point here is that the calorific value of IBAT is consistent with the body’s OS. The hypothalamus is responsible for controlling IBAT activity and is controlled primarily by the sympathetic nerves and body temperature control centers. Oxidation of FFA in IBAT provides the fuel needed for noradrenaline to activate lipolysis and heat production. However, since IBAT uses a lot of O_2_ to generate heat, part of the oxygen molecules are converted to free radicals, especially O_2_^•−^ by mitochondrial assemblies I and III [72]. However, WAT works more to maintain the balance of energy homeostasis and the source of fat storage and release. WAT content includes subcutaneous WAT (scWAT), visceral WAT (vWAT), and peripheral arteries, each containing fat such as the omental, gonadal, retroperitoneum, epicardial, mesenteric, and perineal. However, it should be noted that all of these reserves are responsible for regulating total energy homeostasis [16]. Most importantly, the endocrine properties of AT are embedded in WAT [66]. This means that there are many hormonal mediators, including cytokines (IL-1β, IL-6, and TNFα), adipokines (adiponectin, resistin, and leptin), and chemokines (macrophage inflammatory protein 1 (MIP1), monocyte chemoattractant protein-1 (MCP-1)), ROS and FFA from WAT [73]. However, various studies have shown that pro-inflammatory cytokines are much less expressed in scWAT than in vWAT [29].

On the other hand, different hormones affect the storage and release of WAT fat. For example, after a meal, TG storage is highly dependent on insulin action. While in fasting and when different body organs (skeletal muscle and liver) need energy, the TG stored in WAT is broken down by catecholamines into FFA and G [65]. Furthermore, beige or brown (brown-in-white) fat cells are a new type of exothermic fat cell within WAT. However, these cells have both white and brown fat cells at the same time. They are more similar to fat white cells in terms of growth and more similar to BAT in terms of function and morphology [64,74].

## 6. Metabolic Disorders in AT

One of the practical and important factors in maintaining animals’ and humans’ body weight is maintaining fat homeostasis in WAT. This depends a lot on the proper and adequate performance of the WAT-derived materials [75,76]. Excess fat due to the long-term balance of positive energy contributes to OS in adipocytes, obesity, and subsequent obesity-related metabolic disorders such as hyperglycemia, insulin resistance and cardiovascular disease. [55,65]. Thus, systemic OS is closely related to obesity. During obesity, the concentration of OS indicators such as high-sensitivity C-reactive protein (CRP) and oxidized LDL increases [33]. Obesity results from increasing the size and volume of body fat cells and has adverse effects on the health of living organisms [76,77]. On the other hand, the mismatch between height and body weight due to excessive fat accumulation is named obesity [78]. Today, the people of developed countries are facing obesity-related health problems, but the people of developing countries are grappling with this global dilemma [79].

It is estimated that by 2030, half of all retirees in the United States will suffer from chronic obesity and related diseases, especially cardiovascular disease. This has numerous negative effects on the global health system due to the costly and time-consuming treatment of obesity [80]. Countless BMI studies have accepted more than 30 as obesity, but several studies have shown that BMI is not a good indicator of obesity. These studies cited the inability of this criterion to measure lean mass. These studies have shown that the measure of waist-to-pelvis or waist circumference may be a more accurate and accurate indicator of BMI in calculating fat distribution [16,81]. According to statistics published by the WHO, the prevalence of this disease is likely to reach more than one billion people in the world by 2030, which is a worrying statistic [79,82]. On the other hand, there is a direct and positive relationship between BMI and indicators of oxidative damage to proteins (advanced oxidation protein products (AOPP), lipids (MDA or 8-iso-PGF2α), and DNA (8-OHdG) during obesity) [30].

Oxidative DNA damage due to prolonged exposure to OS impairs mitochondrial function and leads to excessive fat accumulation and subsequent insulin resistance [16]. In recent years, obesity has become widespread due to lifestyle changes such as increased consumption of nutrients, especially fast foods, and decreased physical activity (environmental factors). Of course, the role of genetics is also felt to some extent in the development of this disease, but its effect is not as tangible as environmental factors [83]. There should be a strong emphasis on body weight control as a practical way to prevent obesity-related diseases. This usually happens with lifestyle modifications and focuses on eating healthy and adequate nutrients and engaging in regular exercise [84]. Various studies have shown that obesity is associated with increased OS, decreased antioxidant activity, and insulin resistance in AT [85]. Numerous studies have shown that fat accumulation in obesity is closely related to an increase in ROS and subsequent OS. Therefore, obesity-induced OS plays an important role in disrupting adipokines regulation and amplifying inflammatory signals and even leads to changes in cellular composition and premature aging [84]. It is estimated that with excessive consumption of nutrients, WAT expands (size increases) by 10% per year, which is known as fatty remodeling and the penetration of immune cells into AT [21]. AT remodeling leads to the rapid spread of obesity, which is usually accompanied by changes in the size (hypertrophy) and number (hyperplasia) of fat cells [16,51].

Obesity is the cause of various other diseases such as T2D, dyslipidemia, cardiovascular disease, atherosclerosis, and hypertension [53]. Clinical studies have also shown that BMI is directly related to OS by-products such as protein carbonylation products or lipid peroxidation. By causing OS in AT, adipocytokines’ secretion (IL-1β, IL-6, and TNFα) is disrupted and eventually leads to obesity and associated diseases [85]. Of course, the function of other cells and tissues in the body, including beta pancreatic cells, vascular endothelial cells, and myocytes, is affected by obesity due to adipose tissue [21].

## 7. Antioxidants

As mentioned earlier in this article, oxidative damage can be defined as an imbalance between ROS production and antioxidant defense, leading to overproduction of ROS. The result of this imbalance is a change in cellular redox status. In vivo, antioxidant defense systems play an important role in restoring cellular redox status, especially under normal and stress-free conditions [86]. To combat OS, the body uses enzymatic antioxidant systems (SOD, CAT, peroxidase (POD), peroxiredoxin (Prxs), and glutathione peroxidases (GPX)) and non-enzymatic (carotenoids, tocopherol, and ascorbic acid) [85]. When the body is under pressure using various stressors, especially fat accumulation in AT, these antioxidant defenses alone may not be sufficient and require the use of antioxidant supplements [87,88,89,90,91].

### 7.1. SOD

McCord and Fridovich, by discovering SOD, showed that this enzyme could defend cells exposed to O_2_ as a defense mechanism [86]. To counteract O_2_^•−^, SOD is the first enzyme to convert this free radical to H_2_O_2_ [92,93,94]. Based on specific cofactors and cell locations, there are three different isoforms of SOD. These isoforms include cytosolic (SOD1 or Cu/ZnSOD), mitochondrial SOD (SOD2 or MnSOD), and extracellular SOD (SOD3 or ecSOD) [16].

SOD1 consists of both copper and zinc ions, which are responsible for maintaining enzymatically active sites. SOD1 does this by working with the remaining imidazolate ligands of the histidine SOD1. On the other hand, zinc ions are responsible for stabilizing enzymes in different cells of the body. It has also been estimated that the nuclear part of mammalian cells, cytoplasm, peroxisomes, lysosomes, chloroplasts, and cytosols host SOD1. However, the highest SOD1 activity has been reported in the human liver [91]. The second cofactor of SOD is MnSOD, which has the most increased activity in the renal cortex, and mainly peroxisomes and mitochondrial matrix are the enzyme sites [95]. The third cofactor (EC-SOD) is also present in human lymphocytes and plasma. Zinc and copper are found in this enzyme and effectively remove O_2_^•−^ from tissues [96].

### 7.2. GPx

GPx is usually in the mitochondria and cytosol of various cells and is mainly a glycoprotein containing selenocysteine residues. This antioxidant enzyme is skilled in converting H_2_O_2_ to water. This enzyme also participates in the catalysis cycle to reduce hydroperoxides to alcohol and ultimately involves the oxidation of GSSG induced by GSH [97]. There is a positive relationship between increased GPX concentration and anti-inflammatory activity of the cardiovascular system. On the other hand, lipid hydroperoxides such as cholesterol, free fatty acids, cholesterol esters, and phospholipids are rapidly neutralized by phospholipases and GPX. It is also noteworthy that the detoxification of lipid hydroperoxides is performed by the enzymes PRx, glutathione S-transferase (GST), and GPX [92]. To date, approximately five different isoforms of GPX have been identified. These isoforms include cytosolic or classical GPX (cGPX or GPx1), gastrointestinal GPX (GIGPX or GPX2), plasma GPX (PGPX or GPX3), phospholipid GPX (PHGPX or GPX4), and sperm nuclear GPx or GPx (Sn) [97].

### 7.3. CAT

The peroxisome part of many cells contains the enzyme CAT, which effectively reduces hydrogen peroxide to water. As mentioned earlier, both CAT and GPX are sensitive to H_2_O_2_. These two enzymes are exposed to high and low H_2_O_2_ concentrations, respectively. For this reason, the concentration of free radicals determines the importance of the two enzymes GPX and CAT [98].

Another way to measure ROS is to observe changes in the antioxidant defense system. Tools such as Total Antioxidant Status (TAS), Trolex Equivalent Antioxidant Capacity (TEAC), Total Radical Trapping Antioxidant Parameter (TRAP), Plasma Iron Reduction Capacity (FRAP), and Radical Oxygen Absorption Capacity (ORAC) can measure antioxidant capacity [41].

## 8. Obesity, OS, and Antioxidant Supplementation

Because obesity is more associated with physical inactivity and overeating, genetics play a very limited role in causing the disease. Therefore, for the treatment of obesity, special attention should be paid to the lifestyle because this disease can be prevented and even treated by lifestyle modification [73,99,100]. As mentioned, one of the most important treatment strategies and, of course, prevention of various diseases, especially obesity, is exercise. Multiple studies have shown that AT reserves are reduced by regulating exercise-induced lipase regulation, which ultimately leads to weight loss and obesity treatment. Another important role of exercise is to create antioxidant profiles, which can be a key solution to further reduce body fat due to OS [61]. The results of various studies show the depletion of both enzymatic and non-enzymatic antioxidant systems. However, the type of tissue and the degree of obesity is among the factors that play an important role in the rate of discharge of these systems [30].

These enzymes protect the body’s cells by catalyzing free radicals into water. Various studies have shown that Prxs expression in humans and obese animals is closely related to OS induced by AT. The activity of this enzyme decreases with obesity. On the other hand, PRDXS in adipocytes can increase and decrease lipolytic and lipogenic gene expression, respectively [101]. Scientists have studied the effects of antioxidant supplements on the improvement of obesity caused by OS in various studies. These studies’ results are contradictory, and further studies in this field are still required to reach a correct and logical conclusion. For example, vitamin E is one of the supplements for which the usefulness or harmfulness in treating obesity or other metabolic disorders remains unclear [30].

Simán et al. (1996) examined the effect of consuming an antioxidant diet containing butylated hydroxytoluene (BHT 0.5% and 1%) with or without vitamin E acetate (4%) for four weeks in 30 female Sprague Dawley rats. They concluded no change in the alpha-tocopherol concentration of abdominal AT with BHT supplementation [102]. In another study, Rodrigues et al. (2020) examined the effect of consuming an antioxidant fruit called chestnut at a dose of 1.1% in 18 FVB/Nn male 7-month-old mice. They concluded that this supplement reduced adipose tissue, serum cholesterol, and adipose tissue deposition [103].

Furthermore, Candiracci et al. (2014) investigated the effect of consuming an antioxidant source of rice bran enzymatic extract for 20 weeks in obese and lean Zucker rats. This study’s results included the reduction of overproduction of IL-6, TNF-α, IL-1β, and NOS in abdominal and epidermal visceral AT. In addition, reducing the adipocyte size of abdominal and epidural visceral AT was another effect of this supplement on AT [29]. In a study, Valls et al. (2003) investigated the impact of eating a diet rich in corn oil with or without antioxidant supplementation of vitamin E (30 mg per day) on the antioxidant status and oxidative damage of AT in male Wistar rats. This study showed that the activity of the antioxidant enzymes CAT and SOD was reduced by taking a hyperlipidemia supplement along with vitamin E in AT [104].

In one study, Arias et al. (2014) examined the effect of quercetin (30 mg/kg body weight) in 28 male Wistar rats. This study shows that this supplement has no impact on reducing AT size and body weight. The activity of lipoprotein lipase and lipogenic enzymes remained unchanged with the use of this supplement [105]. Chen et al. (2020) investigated the effect of antioxidant supplementation of protease A-digested crude-chalaza hydrolysates (CCH-As) on Syrian male Golden Hamsters. They showed that adipose-perinatal/hepatic tissue size decreased as a result of consuming this antioxidant composition. Increased lipolysis (unpaired carnitine palmitoyltransferase 1, hormone-sensitive lipase, and protein 2) was also observed in these hamsters’ AT [106]. Because mice, unlike humans, can endogenously synthesize vitamin C (ascorbate and ascorbic acid) and meet their daily needs, it is hypothesized that consuming extra amounts of vitamin C will counteract the anti-inflammatory effects. Therefore, in a study, researchers examined the effect of 4 weeks of vitamin C supplementation (low and high doses of 0.75 and 25 mg of ascorbic acid per kg of body weight, respectively) on male Wistar rats. Excessive consumption of this antioxidant supplement was able to strengthen antioxidant defenses (MnSOD, CuZnSOD, and CAT in AT [107]. Sung et al. (2012) investigated the effect of antioxidant supplementation of Polygonum aviculare L. (knotgrass) (PAE) in male C57BL/6J mice. They were given a high-fat diet or a high-fat diet with PAE antioxidant supplementation at a dose of 400 mg/kg body weight per day. In this article, the researchers found that adipose tissue weight, serum TG concentration, body weight, MDA and leptin concentrations, and fat cell area decreased as a result of taking this supplement [108]. Furthermore, Alcalá et al. (2015) examined the effect of taking antioxidant vitamin E supplementation (150 mg twice daily) in C57BL/6J mice. This study’s results included a reduction in collagen deposition and OS in rat visceral AT. Consumption of this vitamin also led to increased storage capacity and fat cells’ proliferation [30] (Table 1).

## 9. AT, Coronavirus Disease 2019 (COVID-19), and Antioxidants

**Figure 2:**
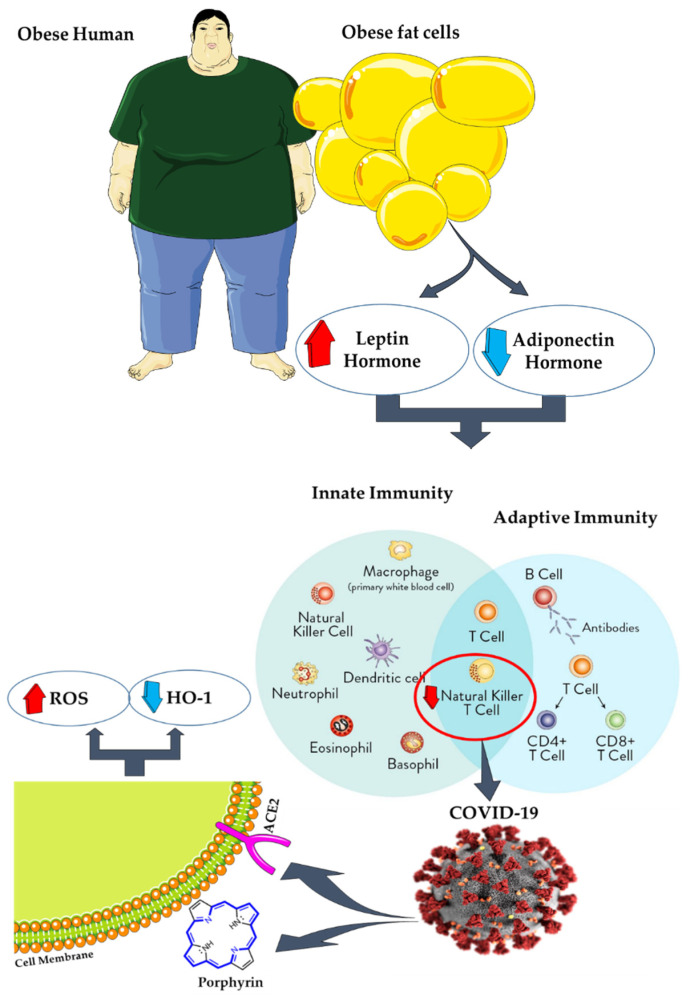
During obesity, adipose tissue (ATs) function is impaired, and secretion of leptin and adiponectin increases and decreases, respectively. Moreover, the immune system’s function is impaired. One of the consequences of an impaired immune system is the induction of viral infections such as COVID-19 due to reduced natural killer (NK) cell activity. COVID-19 requires binding to the Angiotensin-Converting Enzyme 2 (ACE2) receptor and porphyrins on the cell surface to enter and then infect fat cells. Eventually, heme oxygenase-1 (HO-1) and ROS levels decrease and increase, respectively [109,110,111,112,113].

AT is one of the essential tissues that modulate innate and adaptive immune responses in the body. This tissue modulates these responses by secreting adipokines such as leptin and adiponectin. However, during obesity, the function of this tissue is impaired. This means that the secretion of leptin and adiponectin increases and decreases, respectively, and eventually, the immune system’s role is impaired [109]. In such cases, the chest wall is also affected by fat accumulation and impairs the lungs’ proper functioning [110]. One of the consequences of an impaired immune system is the induction of inflammatory cytokines and the development of viral infections such as COVID-19 due to reduced natural killer (NK) cell activity. This infectious disease is caused by SARS-COV-2 (Severe Acute Respiratory Syndrome Coronavirus 2) (Figure 2) [111,112,113].

COVID-19 was first seen in December 2019 in Wuhan Province, China. Then, in January 2020, the disease’s first cases were reported outside China (one in Japan and two in Thailand). Since then, the disease has spread rapidly to all countries of the world [114,115]. The condition was declared a pandemic on 11 March 2020, by WHO on 11 March 2020, and to date (22 February 2021), the total number of infected patients has reached 112,045,556, of which 2,479,625 people lost their lives (https://www.worldometers.info/coronavirus/, accessed on 20 February 2021). The virus requires binding to the Angiotensin-Converting Enzyme 2 (ACE2) receptor and porphyrins on the cell surface to enter and then infect fat cells. Eventually, heme oxygenase-1 enzymes (HO-1) and ROS levels decrease and increase, respectively [116,117,118].

Fatigue, headache, fever, and loss of taste and smell are symptoms associated with this disease, and most of these infected people recover without hospitalization. Various studies examining healthy people and people with underlying conditions have shown that people with cardiovascular disease, kidney damage, diabetes, and severe obesity (BMI ≥ 30 kg/m^2^) are more susceptible to the virus [119]. The risk of developing COVID-19 does not depend on age, and the severity of the disease follows a different pattern at each age. According to Public Health England (PHE), the risk of COVID-19 death in people with a BMI between 35 and 40 kg/m^2^ increases by 40%. However, this increase of risk in people with a BMI ≥ 40 kg reaches 90% [120]. As mentioned, obesity is directly related to COVID-19 disease and leads to increased inflammation, mitochondrial dysfunction, and increased ACE2 receptors. Numerous studies have shown that high BMI (≥30 kg/m^2^) and excess visceral fat (VF) are effective methods in diagnosing the severity of COVID-19, especially in obese patients [115,119,121].

For more than a year, the COVID-19 disease has affected human society in all aspects of life. The medical community has been able to develop effective vaccines against the disease. Furthermore, scientists in authoritative articles have suggested various drugs and nutrients reduce inflammation in the immune system, indirectly helping cure the disease. Among the various nutrients, antioxidants (vitamins C, D, and E, iron, and selenium) have always been at the forefront of strengthening the immune system and reducing inflammation in various body tissues, especially AT [122]. The recommended dose of vitamins C, D, and E in healthy individuals is 200 mg/day, 2000 IU/day (50 µg/day), and 15 mg/day, respectively. However, in patients who have inflammation in their immune system, it is better to increase the daily intake of vitamin C to 1-2 gr. It has also been suggested that the daily dose of vitamin D in these patients be increased to 10,000 IU in the first few weeks and then continued at a dose of 5000 IU. Also, the daily intake of vitamin E in these patients should be increased to 200 IU [123,124]. Consumption levels of another nutrient, iron, are usually about 8 mg daily in men, approximately 18 mg in women between the ages of 19 and 50, and around 8 mg in women over 51 years of age. However, if the person has inflammation in the immune system, 60 mg Fe should be consumed daily in both men and women and all age groups [114]. The daily intake of selenium in healthy men and women is 50 µg, respectively, but in inflammatory conditions, this amount increases to 200 µg per day [125].

## 10. NAFLD and OS

**Figure 3:**
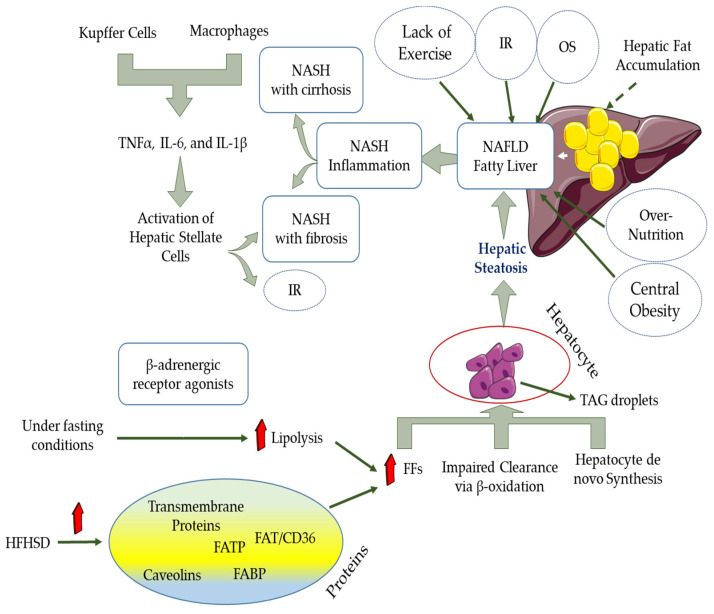
Various factors such as central obesity, insulin resistance (IR), type 2 diabetes (T2D), overnutrition, lack of exercise, and other metabolic syndrome parameters predispose multiple diseases such as NAFLD. Macrophages and Kupffer cells can stimulate pro-inflammatory mechanisms and then satellite cell activity at the liver surface by secreting inflammatory cytokines such as IL-6, TNF-α, and IL-β. In such inflammatory environments, conditions are provided for increasing the deterioration of IR and the development of liver fibrosis. It should be noted that the increased flow of FAs to the liver through the bloodstream, the synthesis of de novo hepatocytes, and impaired clearance through β-oxidation lead to the accumulation of TAG droplets in hepatocytes. Various proteins such as FATP, transmembrane proteins, FABP, caveolins, and FAT/CD36 can accelerate the absorption of FA by increasing the proliferation of FFA in blood vessels. It is noteworthy that these proteins’ expression can improve by an HFHSD. On the other hand, in fasting conditions, FFAs are mainly produced during the lipolysis process by beta-adrenergic receptor agonists [126,127,128,129,130,131,132,133].

Various factors such as central obesity, IR, T2D, over nutrition, lack of exercise, and other metabolic syndrome parameters predispose multiple diseases such as non-alcoholic fatty liver disease (NAFLD) [126,127]. NAFLD is usually characterized by fat accumulation in the liver tissue, and oxidative stress plays a crucial role in its formation and development. It covers a wide range of liver-related diseases such as steatosis, steatohepatitis, liver fibrosis, liver cirrhosis, and even hepatocellular carcinoma [128,129]. For NAFLD, there are non-progressive forms (non-alcoholic fatty liver disease (NAFLD) or simple steatosis) and progressive and aggressive forms (non-alcoholic steatohepatitis (NASH)). Hepatocellular carcinoma (HCC) and cirrhosis are considered as consequences of NASH [130,131,132]. Macrophages and Kupffer cells can stimulate pro-inflammatory mechanisms and then satellite cell activity at the liver surface by secreting inflammatory cytokines such as IL-6, TNF-α, and IL-β. In such inflammatory conditions, conditions are provided for increasing the deterioration of insulin resistance and the development of liver fibrosis (Figure 3) [133]. In general, in patients with NAFLD, lipids’ storage capacity in the liver tissue is so high that it leads to hepatocyte dysfunction and even death [134].

Numerous studies have shown that the mechanisms associated with the pathogenesis of obesity and NAFLD are the same [135]. A two-hit theory can usually explain the pathogenesis of NAFLD. The first theory is explained when triacylglycerol (TAG) droplets accumulate in hepatocytes and lead to simple hepatic steatosis development. In the second theory, NAFLD’s pathogenesis is attributed to increased oxidative stress, IR, lipid peroxidation, and endoplasmic reticulum inflammation [136,137]. It should be noted that the increased flow of FAs to the liver through the bloodstream, the synthesis of de novo hepatocytes, and impaired clearance through β-oxidation lead to the accumulation of TAG droplets in hepatocytes. TG synthesis in the liver is mainly due to the lipids produced by de novo lipogenesis (DNL), dietary lipids, and carbohydrates. TG synthesis is dependent on the uptake of FFAs from the plasma by the liver [138]. DNL is the process by which exogenous energy sources or endogenous carbohydrates can synthesize lipids. Three steps are defined for this process. First, FAs can be synthesized through acetyl-CoA subunits produced during glycolysis and carbohydrate metabolism. Then, to form long-chain unsaturated FAs, FA elongation and desaturation must occur. Finally, the FAs formed from the previous step are assembled to convert to TG and very-low-density lipoproteins (VLDLs). When the balance between TG synthesis and degradation is lost, the conditions for NAFLD are created [130]. Various proteins such as fatty acid (FA) transporter protein (FATP), transmembrane proteins, FA binding protein (FABP), caveolins, FA translocase (FAT)/CD36 can accelerate the absorption of FA by increasing the proliferation of FFA in blood vessels. It is noteworthy that these proteins’ expression can improve by a high-fat, high-sugar diet (HFHSD). On the other hand, in fasting conditions, FFAs are mainly produced during the lipolysis process by beta-adrenergic receptor agonists [139].

AT is severely affected by NAFLD because it is a source of FAs storage, and the secretion of adipokines is impaired. AT acts like a double-edged sword. This means that some hormones secreted by AT, such as adiponectin and visfatin, have protective effects against NAFLD; however, the hormones resistin and leptin contribute to hepatic development of steatosis and IR [140,141].

ROS production in hepatic mitochondria results from excessive oxidation of fatty acids, which ultimately causes OS in liver tissue. Proteins, DNA, and lipids are susceptible to OS and are easily damaged by activating pro-inflammatory cells such as Kupffer cells and stimulating the release of inflammatory cytokines. Furthermore, the expression and activity of antioxidant enzymes are usually inhibited by ROS overload, and thus, the liver’s antioxidant capacity undergoes a declining trend. Finally, NAFLD occurs as a result of OS and chronic inflammation. Researchers should try to reduce OS to improve NAFLD disease [142].

Approximately 25% of adults worldwide are affected by this disease. This trend is increasing, and the number of these patients increases every year. According to a meta-analysis study, the global prevalence of NAFLD has risen to 25.2% in the last 20 years and has caused concern among the public [143]. On the other hand, no effective treatment for this disease has been achieved despite significant medical advances. Currently, the only treatment approach is lifestyle changes (diet and exercise) and bariatric surgery [144]. Because there are substantial differences between different communities in terms of lifestyle and diet, various studies have shown that the prevalence of NAFLD in Eastern societies is lower than in Western societies [145]. At the systemic level, there is impaired control of food intake resulting in hyperalimentation, intestinal dysbiosis leading to gastrointestinal hormone secretion, IR, gut dysfunction, abnormal adipokine, and activation of pro-inflammatory factors [146].

One of the effective strategies in the prevention and treatment of NAFLD is nuclear factor erythroid-derived 2-like 2 (Nrf2), which as a transcription factor consists of a highly protected basic region-leucine zipper (bZIP) structure and is mainly a member of the Cap “n” Collar (CNC) family. Activation of cellular antioxidant enzymes, regulation of lipid metabolism, and insulin sensitivity improvement are the essential cytoprotective effects of Nrf2. Hence, many researchers have tried to identify Nrf2 activators to improve NAFLD [147].

The Kelch-like-ECH-associated protein 1 (Keap1)-Nrf2-antioxidative response element (ARE) signaling pathway has been considered an essential antioxidant mechanism due to its effect on improving the oxidative stress response [148]. The Nrf2 gene includes six highly protected epichlorohydrin (EHC) domains (Nrf2-EHC homology, Neh) called Neh1-6. The C-terminus Neh1 subtends a protected bZIP DNA region that binds to musculoaponeurotic fibrosarcoma protein (Maf) to create a heterodimer. This heterodimer eventually binds to DNA and can detect ARE. On the other hand, Neh2 is composed of two vital regions, ETGE and DLG, which, by binding to KEAP1, can contribute to the strong binding of Nrf2 to the cytoplasm [129]. C-terminus is the site of another Nrf2 domain, Neh3, which participates in the transcriptional activity of ARE after binding with chromo-ATPase/helicase DNA-binding protein (CHD6). The other two Nrf2 domains, Neh4 and Neh5, initiate the transcription process when interacting with the cyclic adenosine monophosphate response element (CREB)-binding protein (CBP) [149]. Finally, the last Nrf2 domain, Neh6, and being rich in serine are used to Nrf2 decompose independent of KEAP1 [150]. The expression of Nrf2 in homeostatic conditions and combination with KEAP1 in the cytoplasm is considered a mediator for the degradation and ubiquitinoylation of Nrf2. However, when exposed to oxidative or electrophilic stress, KEAP1 modulates cysteine residues and ultimately releases Nrf2. On the other hand, the Nrf2 protein isolated from KEAP1 returns to the cell nucleus and is dimerized to bind to AREs, along with bZIP proteins such as Maf [151], and then promotes the expression of ARE-mediated downstream target genes containing antioxidant enzymes. The most critical antioxidant proteins targeting Nrf2 are HO-1, GSH, and NAD(P)H quinone oxidoreductase 1 (NQO1). It should also be noted that Nrf2 plays a pivotal role in suppressing the progression of NAFLD, maintaining cellular homeostasis, and protecting against oxidative or electrophilic stresses [152].

## 11. Roles of Nutraceuticals as an Antioxidant in Reducing Oxidative Stress

Nutraceuticals are not recognized traditionally as a nutrient but have physiological health benefits in the human body. Plant-derived nutraceuticals are well-known for their direct or indirect antioxidant activities, which relates to scavenging or eliminating free radicals during cellular metabolism. They can interact with the oxidized species at both cellular and molecular levels by regulating gene expression, epigenetic controls, and protein and DNA repair. Previously it was reported that nutraceuticals have potential properties in immunity modulation, gene expression, and various signaling process regulation [153,154,155,156,157,158]. The nutraceuticals can be prepared from the foods available in the local market, for example, ginger, garlic, avocado, and onion, in the form of polyphenols, carotenoids, sulforaphane and other isothiocyanates, glucosinolate, phytosterol, etc. [159]. They can increase the level of heme oxygenase (HO) 1, total glutathione, and other phase 2 enzymes by activating the transcription Nrf2. Moreover, treatment for a certain period with nutraceuticals may also improve the lipid profile and can reverse the harmful effects of obesity on blood lipids [160]. For example, curcumin is a potential nutraceutical, reduces macrophage infiltration in WAT, increase adiponectin in AT, decreases NF-κB activity, therefore reduces the expression of inflammatory markers and OS [161]. To date, the use of nutraceuticals, bioactive compounds or exercise could be an additional strategy in reducing obesity and related diseases [162,163].

## 12. Conclusions

OS affects various tissues, such as adipose tissue, skeletal muscle, and heart, in the body. In this study, we specifically examined adipose tissue response to OS. As mentioned in the text, this tissue is disrupted by various factors such as overconsumption of nutrients and sedentary lifestyle. This disorder eventually leads to lipid accumulation in adipose tissue and reduced energy expenditure. Of course, various treatments have been introduced for this disorder. However, most of them face limitations that are fully explained in the text. On the other hand, numerous studies have proven the effectiveness of diet, especially the use of antioxidant supplements, on the improvement of obesity caused by OS. The results of this treatment are inconsistent but have fewer side effects than other treatments such as medication and surgery. Further studies are needed because the results of the studies are contradictory. In future studies, researchers will investigate the effect of taking antioxidant supplements on heart and skeletal muscle tissues.

## Figures and Tables

**Table 1:** The effect of antioxidant supplementation on obesity caused by oxidative stress (OS).

Reference	Subjects	Antioxidant Supplementation	Results
Simán et al. [102]	Sprague Dawley rats	BHT (0.5% and 1%) with or without vitamin E acetate (4%) for four weeks.	No change in the alpha-tocopherol concentration of abdominal AT with BHT supplementation.
Rodrigues et al. [103]	FVB/n male 7-month-old mice	Chestnut at a dose of 1.1%.	The reduction of serum cholesterol and AT deposition.
Candiracci et al. [29]	Obese and lean Zucker rats	Rice bran enzymatic extract (RBEE) for 20 weeks.	The reduction of overproduction of IL-6, TNF-α, IL-1β, and NOS in abdominal and epidermal visceral AT. Reducing the adipocyte size of abdominal and epidural visceral AT.
Valls et al. [104]	Male Wistar rats	Diet rich in corn oil with or without antioxidant supplementation of vitamin E (30 mg per day).	The reduction of activity of the antioxidant enzymes CAT and SOD.
Arias et al. [105]	Male Wistar rats	Quercetin (30 mg/kg body weight).	No impact on reducing AT size and body weight. No change in the activity of lipoprotein lipase and lipogenic enzymes.
Chen et al. [106]	Syrian male Golden Hamsters	Protease A-digested crude-chalaza hydrolysates (CCH-As).	The reduction adipose-perinatal/hepatic tissue size. The increase of lipolysis (unpaired carnitine palmitoyltransferase 1, hormone-sensitive lipase, and protein 2).
Djurasevic et al. [107]	Male Wistar rats	Vitamin C supplementation (low and high doses of 0.75 and 25 mg of ascorbic acid per kg of body weight, respectively) for 4 weeks.	Excessive consumption of this antioxidant supplement was able to strengthen antioxidant defenses (MnSOD, CuZnSOD, and CAT in AT
Sung et al. [108]	Male C57BL/6J mice	High-fat diet or a high-fat diet with PAE at a dose of 400 mg/kg body weight per day.	The reduction of AT weight, serum TG concentration, body weight, MDA and leptin concentrations, and fat cell area.
Alcalá et al. [30]	C57BL/6J mice	Vitamin E supplementation (150 mg twice daily).	The reduction in collagen deposition and OS in rat visceral AT. The increase of storage capacity and fat cells’ proliferation.
